# Neck Management in Malignant Parotid Tumors: A Retrospective Analysis of Elective Neck Dissection Indications and Outcomes

**DOI:** 10.3390/diagnostics15243194

**Published:** 2025-12-14

**Authors:** Andrea Battisti, Giulio Pagnani, Giulia Scivoletto, Marco Della Monaca, Matteo Fatiga, Andrea Cassoni, Valentino Valentini

**Affiliations:** Department of Odontostomatological and Maxillofacial Sciences, Sapienza University, 00185 Rome, Italymarco.dellamonaca@uniroma1.it (M.D.M.); matteo.fatiga@uniroma1.it (M.F.);

**Keywords:** parotid neoplasms, neck dissection, lymphatic metastasis, intraparotid lymph nodes, head and neck oncology

## Abstract

**Background/Objectives:** Management of the clinically negative neck in malignant parotid tumors remains controversial. We aimed to identify clinicopathologic predictors of nodal involvement and to evaluate whether elective neck dissection (END) improves disease-free survival (DFS) versus observation in cN0 patients. **Methods:** We performed a retrospective cohort study of adults undergoing surgery for malignant parotid tumors at a single tertiary center (2013–2023) with ≥24 months of follow-up. Collected variables included demographics, tumor T category and histologic grade (AJCC 8th), parotidectomy type, neck management [END vs. therapeutic neck dissection (TND) vs. observation], lymph node yield, and outcomes. Associations were tested with Fisher’s exact tests; disease-free survival (DFS) was analyzed using Kaplan–Meier curves, log-rank tests and an exploratory multivariable Cox proportional hazards model. **Results:** Seventy-four patients were included (mean age 54.3 years; 12.2% preoperative facial nerve impairment). Parotidectomy was partial (41.9%), total (31.1%), radical (21.6%), or extended (5.4%). Neck dissection was performed in 40.5% (END 23.0%; TND 17.6%). Overall pathologic nodal positivity (pN+) was 18.9%. T3–T4 tumors had greater odds of nodal metastasis than T1–T2 (OR 10.58; *p* < 0.05). Among cN0 patients, occult metastasis was 17.6%; notably, all high-grade cN0 tumors that underwent END were pN+. Intraparotid nodal metastases occurred in 28.6% and always co-occurred with cervical metastases. DFS did not differ significantly between cN0 patients managed with END versus observation (log-rank *p* > 0.05). Patients with pN0 had superior DFS versus pN+ (*p* < 0.05). Lymph node yield groupings (0–17 vs. 18–40 vs. >40) were not associated with recurrences. In the exploratory multivariable Cox model, high/intermediate-grade and T3-T4 tumors and nodal positivity were associated with reduced DFS. **Conclusions:** Higher T category and high/intermediate grade strongly predict nodal involvement, and pN+ status portends worse DFS. Although END did not show a DFS advantage over observation in cN0 patients, the 17.6% occult metastasis rate—especially in high-grade disease—and the linkage between intraparotid and cervical metastases support a risk-adapted END strategy and intraoperative assessment of intraparotid nodes to guide neck management.

## 1. Introduction

Salivary gland malignancies comprise 0.5 to 1.2% of all cancers and 5% of head and neck cancers, accounting for approximately 15% to 30% of all salivary gland neoplasms [1,2,3]. Major salivary gland cancers (SGCs) account for 5% of head and neck cancers in Europe. The global crude incidence rate is 0.69 cases per 100,000 individuals per year, while the age-adjusted incidence rate stands at 0.57 cases per 100,000 individuals annually [4]. The complexity of these tumors lies in their diverse histological subtypes, including mucoepidermoid carcinoma, adenoid cystic carcinoma, and salivary duct carcinoma and many others, each exhibiting distinct biological behaviors that can significantly impact prognosis and treatment strategies.

According to the National Comprehensive Cancer Network (NCCN) guidelines, the standard treatment of resectable carcinomas of the major glands is surgical excision, on a type 2A category of evidence. Although malignant tumors can arise in various salivary glands, this article focuses specifically on parotid gland tumors, which are the most common and present unique clinical challenges. Parotidectomy is regarded as the optimal method for achieving surgical resection with clear margins. Limited excision or partial resection of the parotid gland is generally discouraged due to the significant risk of incomplete tumor removal. The facial nerve may need to be sacrificed in cases of preoperative facial nerve involvement accompanied by facial palsy, or when there is direct tumor invasion into the nerve that prevents its separation from the tumor [5]. The facial nerve deficit should be addressed immediately by performing a nerve reconstruction.

The management of potential cervical nodal metastases remains a critical and controversial aspect of treatment. Occult neck metastases have been reported in approximately 25% of patients with malignant parotid tumors, underscoring the need for accurate assessment of neck status during treatment planning [6,7]. Despite the recognized impact of nodal disease on prognosis, there is still no consensus on the indications for elective neck dissection (END) in clinically N0 patients. Recent meta-analyses highlight persistent heterogeneity in practice patterns and emphasize the need for standardized, risk-adapted strategies [8].

In this context, there is a clear need for predictive tools that can stratify patients according to their risk of occult nodal metastasis and regional recurrence, thereby informing tailored neck management. The objective of this study is to analyze a single-institution cohort of patients with malignant parotid tumors treated over the last decade, in order to identify clinical and pathological factors associated with nodal involvement and to assess the impact of nodal status on survival. Ultimately, our aim is to provide evidence-based recommendations that may refine decision making for neck management in this complex patient population.

## 2. Materials and Methods

### 2.1. Study Population

All consecutive patients who underwent primary surgery for malignant parotid gland tumors at the Maxillofacial Surgery Unit of Umberto I General Hospital in Rome, Italy, between January 2013 and December 2023 were retrospectively identified from institutional records (*n* = 91). Inclusion criteria were: age ≥18 years, histologically confirmed primary malignant epithelial parotid tumor, and surgery with curative intent. Patients were excluded if they were younger than 18 years, if their follow-up was shorter than 24 months in the absence of recurrence or death, or if clinical and/or pathological documentation was incomplete. After applying these criteria, 74 patients were included in the final analysis. For these 74 patients, all clinical, pathological, and follow-up variables considered in this study were fully available; thus, no additional missing-data handling or imputation procedures were required.

### 2.2. Data Collection and Analysis

Clinical, pathological, and follow-up data were retrospectively retrieved from the institutional electronic medical records by a single author using a standardized data collection form; no independent duplicate data extraction was performed. Preoperative diagnoses were established through various methods, including open biopsy (for large tumors with skin infiltration and ulceration), fine-needle aspiration cytology (FNAC), and fine-needle aspiration biopsy (FNAB), though not every patient underwent these diagnostic procedures prior to surgery. Data collected included patient demographics, tumor characteristics, preoperative facial nerve palsy, surgical procedures, neck dissection details, postoperative histology, adjuvant treatment, and recurrence data. Statistical analyses were performed using Python (version 3.11.13) with the SciPy library (version 1.15.3) [9,10]. Fisher’s exact test (or the Freeman–Halton extension, when appropriate) was used to assess associations between categorical variables, including T-category, histologic grade, histologic subtype, intraparotid nodal status and lymph node yield groups (0–17, 18–40, >40 nodes), and categorical outcomes such as cervical nodal status (pN0 vs. pN+) or recurrence (yes vs. no). Survival analyses were performed using the Kaplan–Meier method and compared using the log-rank test, evaluating disease-free survival (DFS). Time-to-event outcomes were calculated from the date of primary surgery to the date of first recurrence; patients who were alive and recurrence-free at last contact (including those lost to follow-up) were censored at the date of their last clinical or radiological assessment. In exploratory analyses, a Cox proportional hazards model for DFS was fitted including three binary covariates: T-category (T1–T2 vs. T3–T4), histologic grade (low vs. high/intermediate), and nodal status (pN0 vs. pN+). The results reported include the 95% confidence interval (CI), and statistical significance (*p* < 0.05).

### 2.3. Surgical Procedures

Patients underwent one of the following surgical interventions based on malignant tumor characteristics:

- Partial Parotidectomy: This procedure involves the removal of the superficial lobe of the parotid gland while preserving the facial nerve.
- Total Parotidectomy: This procedure consists of a superficial parotidectomy followed by a deep parotidectomy, ensuring the preservation of the facial nerve.
- Radical Parotidectomy: This procedure entails a total parotidectomy with complete removal of the facial nerve, typically necessitated by either clinical evidence of nerve involvement or tumor extension into the nerve pathway.
- Extended Parotidectomy: This technique is applied when there is extensive tumor involvement, necessitating wider resections beyond the confines of the parotid gland to achieve clear margins.

### 2.4. Neck Dissection

Depending on the clinical assessment, patients underwent one of two types of neck dissections:

- Therapeutic Neck Dissection: This procedure is performed when there is clinical or radiological evidence of lymphatic involvement, ensuring thorough excision of affected lymph nodes.
- Elective Neck Dissection: Conducted on patients considered at elevated risk for occult metastatic disease, this approach aims to pre-emptively address potential nodal involvement. Patients considered at elevated risk were those with locally advanced (cT3-T4) tumors and high-grade histology (in this context, high-risk histologies predominantly include squamous cell carcinoma, adenocarcinoma, undifferentiated carcinoma, high-grade mucoepidermoid carcinoma, salivary duct carcinoma, and adenoid cystic carcinoma) [11,12,13].

### 2.5. Pathological Staging

All patients were classified according to the AJCC 8th edition staging system (2017). Data were collected on the number of metastatic lymph nodes, and the proportion of patients with occult nodal metastases, defined as those with clinically negative necks (cN0) preoperatively who were found to have pathologically positive nodes (pN+) following elective neck dissection (END). Total lymph node yield (LNY), defined as the number of lymph nodes retrieved at neck dissection, was recorded for each patient. For analysis, LNY was categorized into three groups: Group A, 0–17 nodes; Group B, 18–40 nodes; and Group C, >40 nodes. The threshold of 18 nodes was chosen in line with published head and neck oncology data indicating this value as the minimum acceptable lymph node yield for an adequate selective neck dissection, thus separating lower-yield (potentially suboptimal) dissections (<18 nodes) from adequately sampled necks (≥18 nodes) [13]. In addition, these cut-offs provided a reasonably balanced distribution of patients across the three categories within our limited cohort, allowing more stable exploratory comparisons between LNY groups and oncologic outcomes. Data on perineural and lymphovascular invasion were also collected.

### 2.6. Follow-Up

Follow-up was carried out according to a uniform institutional protocol. During the first 5 years after surgery, all patients underwent clinical examination every 3 months, with alternating neck ultrasound and contrast-enhanced MRI at each visit to monitor for local and regional recurrence and to estimate disease-free survival. Patients who remained with no evidence of disease (NED) beyond 5 years were subsequently followed with annual clinical assessment and contrast-enhanced MRI. In addition, all patients received annual imaging for distant staging, consisting of either high-resolution CT (HRCT) of the chest or contrast-enhanced CT of the chest and abdomen or PET/CT. HRCT/CT was preferentially used in patients with adenoid cystic carcinoma, given the indolent growth pattern of this histotype and its potential to produce false-negative results on PET/CT. Additional imaging was performed at the discretion of the multidisciplinary team in case of new symptoms or equivocal findings.

## 3. Results

### 3.1. Patient Demographics and Tumor Characteristics

The study cohort comprised 74 patients diagnosed with malignant tumors of the parotid gland. The mean age was 54.3 years, with a male-to-female ratio of 6:7. Clinical facial palsy or impairment was present in 9 patients (12.2%).

### 3.2. Preoperative Diagnostic Procedures

Preoperative diagnostic procedures revealed benign histology in 30.9% of evaluated patients. Notably, not all patients underwent preoperative histological or cytological diagnosis but among patients who had it (75.7%), diagnostic methods included open biopsy in 35.7% of cases, fine-needle aspiration biopsy (FNAB) in 8.9%, and fine-needle aspiration cytology (FNAC) in 55.4%. The accuracy of FNAC was suboptimal, with incorrect diagnoses in 48.4% and non-diagnostic results in 9.7%. Similarly, FNAB showed incorrect diagnoses in 20.0% and non-diagnostic outcomes in 40.0%. In four cases, inconclusive FNAC results necessitated subsequent open biopsy. It is important to note that the high proportion of open biopsies is partly due to the inclusion of all surgical procedures aimed at the excision of a suspected benign tumor, which later turned out to be malignant upon histological analysis and, consequently, required radical intervention (Table 1).

### 3.3. Surgical Management

Surgical management involved partial parotidectomy in 31 patients (41.9%), total parotidectomy in 23 patients (31.1%), radical parotidectomy in 16 patients (21.6%), and extended parotidectomy in 4 patients (5.4%). Neck dissection was performed in 30 patients (40.5%), while 44 patients (59.5%) did not undergo this procedure. Among those who underwent neck dissection, therapeutic neck dissection (TND) was performed in 13 patients (17.6%) and elective neck dissection (END) in 17 patients (23.0%) (Table 2).

We categorized the 30 patients who underwent neck dissection into three classes based on the number of LNY: Group A consisted of 9 patients with 0 to 17 lymph nodes removed, Group B included 11 patients with 18 to 40 lymph nodes, and Group C had 10 patients with more than 40 lymph nodes removed. We conducted three Fisher’s exact tests comparing these groups (A: 0–17 vs. B: 18–40; A: 0–17 vs. C: >40; B: 18–40 vs. C: >40). We observed no statistically significant association between lymph node yield group and recurrences in the patients studied.

### 3.4. Histological Types and Grading

The most common histological types identified were mucoepidermoid carcinoma (MEC) present in 24 patients (32.4%), salivary duct carcinoma in 13 patients (17.6%), acinic cell carcinoma in 6 patients (8.1%), squamous cell carcinoma (SCC) in 6 patients (8.1%), and adenoid cystic carcinoma (AdCC) in 7 patients (9.5%) (Table 3).

In terms of tumor grading, using standard schemes in a 3-tier manner, high/intermediate-grade tumors accounted for 46 patients (62.2%), low-grade tumors for 20 patients (27.0%), and 8 patients (10.8%) had an unknown or unspecified tumor grade (Table 4).

### 3.5. Pathological Features and Nodal Metastasis

Pathological features showed T-classification as T1/T2 in 48 patients (64.9%) and T3/T4 in 26 patients (35.1%). Pathological nodal involvement (pN+) was identified in 14 patients (18.9%). Obviously, pN+ was significantly more frequent in the TND group (92.0%) compared to the END group (17.6%). Among the pN+ cohort, 78.6% of tumors were classified as T ≥ 3. The Fisher exact test demonstrates a highly significant association between T stage (T1/T2 vs. T3/T4) and Nodal metastasis, with a *p*-value of 0.0003, moreover T3/T4 tumors have approximately 10.58 times higher odds of having positive lymph nodes compared to those with T1/T2 tumors., 95% Confidence Interval (2.38 to 66.81).

In our cohort occult metastasis rate in patient cN0 was 17.6%. Occult metastasis was observed in 100.0% of high-grade tumors (out of the 3 cN0 high-grade patients, 3 were found pN+ after the END). Intraparotid positive nodes were identified in 28.6% of patients, all of these patients also had cervical nodal metastasis.

The rates of nodal metastasis (N+) for different histological subtypes included undifferentiated carcinoma at 100.0%, salivary duct carcinoma at 46.2%, SCC at 33.3%, secretory carcinoma at 25.0%, and MEC at 12.5%. The association between histological subtype and nodal metastasis was significant, as confirmed by Freeman-Halton extension of Fisher’s exact test executed for the four most common histologies (*p* < 0.05).

For grading categories, N+ was present in 85.7% of high/intermediate-grade tumors and in 14.3% of low-grade tumors. Our statistical analysis revealed a significant correlation, through Fisher’s exact test, between tumor grade and nodal involvement (*p* = 0.0096), further underscoring the importance of tumor characteristics in predicting metastatic risk.

Perineural invasion was observed in 16 patients (21.6%), while lymphovascular invasion was present in 11 patients (14.9%) (Table 5). Among patients with preoperative facial palsy or impairment, 66.0% had N+.

**Figure 1:**
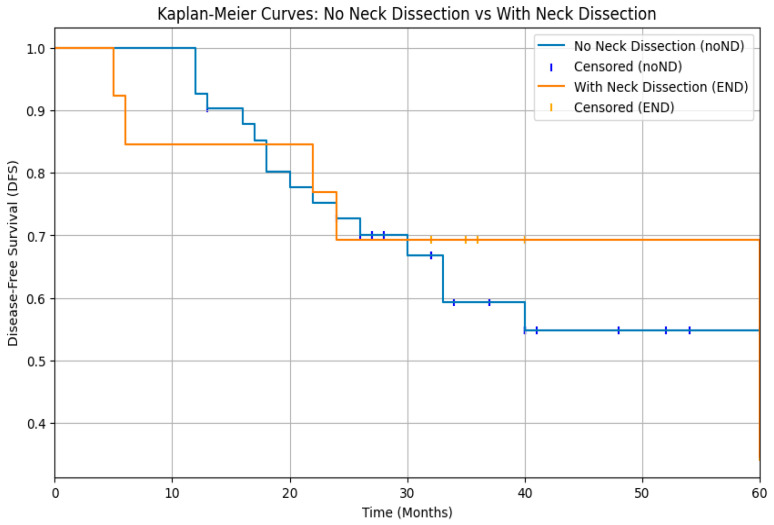
Kaplan–Meier curves for disease-free survival (DFS) in cN0 patients managed with elective neck dissection (END) versus observation. No significant difference in DFS was observed between the two groups (log-rank *p* > 0.05).

**Figure 2:**
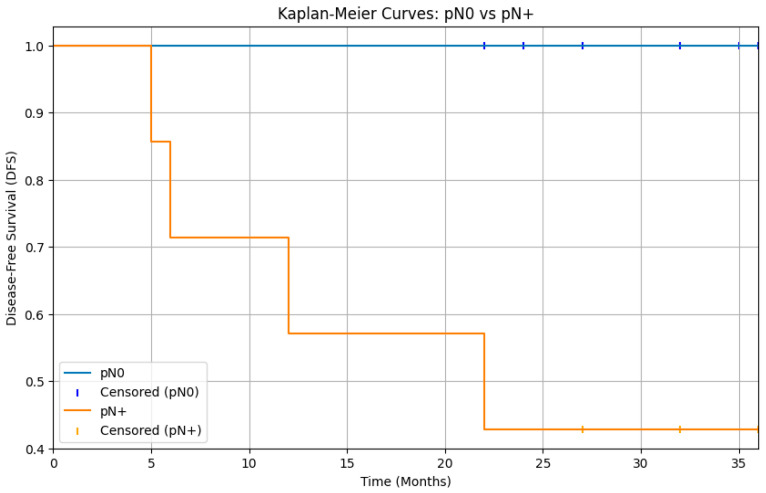
Kaplan–Meier curves for disease-free survival (DFS) comparing patients with pathologically negative lymph nodes and patients with pathologically positive lymph nodes. The curves show a separation, with the pN0 group having significantly better DFS than the pN+ group. The log-rank test results indicate a statistically significant difference in disease-free survival between patients with pN0 and pN+ status (*p* < 0.05).

The analysis of survival rates through log-rank test of DFS Kaplan–Meier curves showed that cN0 patients who underwent elective neck dissection (END) did not experience a significant benefit compared to those who were merely observed (*p* > 0.05) (Figure 1). Moreover, a statistically significant difference was observed between patients with pathologically negative lymph nodes (pN0) and those with positive lymph nodes (pN+), with a clear survival advantage in patients without laterocervical metastases (*p* < 0.05) (Figure 2).

**Figure 3:**
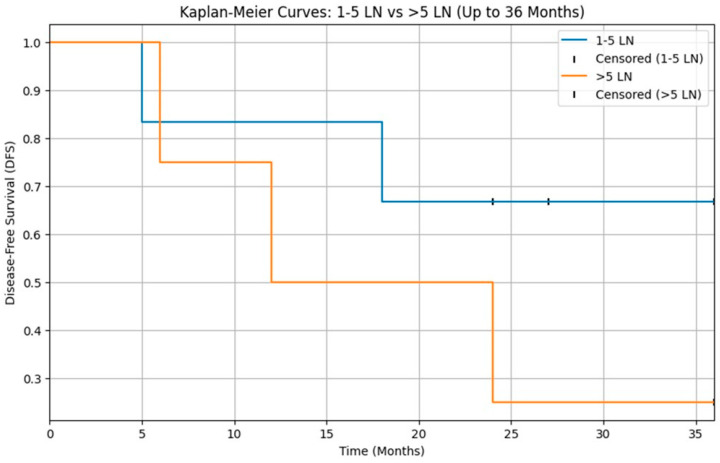
Kaplan–Meier curves for disease-free survival (DFS) according to nodal burden in pN+ patients (1–5 vs. >5 positive lymph nodes). DFS did not differ significantly between the two groups (log-rank *p* > 0.05).

Lastly although DFS appeared lower in patients with >5 positive lymph nodes compared with those with 1–5 positive nodes, this difference did not reach statistical significance (log-rank *p* = 0.05), likely reflecting the very small number of pN+ patients (*n* = 14) and the limited power of the analysis (Figure 3).

### 3.6. Recurrences

The median follow-up for the cohort was 52 months. In total, 81.1% of patients completed the intended 5 years of follow-up, whereas 18.9% were lost to follow-up; most losses (14.9% of the entire cohort) occurred after the third year of follow-up.

Recurrences were observed in 29.7% of patients, with T recurrence in 13.5%, N recurrence in 18.9%, and M recurrence in 17.6%. The median interval to recurrence was 24 months. Among those tumors that had N recurrences, 71.4% were classified as high/intermediate grade.

In an exploratory multivariable Cox proportional hazards model for DFS including T-category, histologic grade, and nodal status, all three variables remained associated with disease outcomes. High/intermediate-grade tumors were associated with worse DFS compared with low-grade tumors (HR 1.8, 95% CI 1.4–2.3; *p* < 0.05). Advanced T-category (T3–T4 vs. T1–T2) was also an adverse factor (HR 2.0, 95% CI 1.5–2.8; *p* < 0.05), as was nodal positivity (pN+ vs. pN0; HR 2.4, 95% CI 1.7–3.2; *p* < 0.05). Given the small number of DFS events (*n* = 22), these multivariable estimates are considered exploratory and require validation in larger series.

## 4. Discussion

This study presents a detailed analysis of malignant parotid tumors treated at our institution over a 10-year period, providing valuable insights into patient demographics, diagnostic challenges, surgical management, and prognostic factors. Our findings, while limited by the small sample size due to the rarity of the disease, offer a unique perspective that complements and contrasts with existing literature.

The mean age and gender distribution in our cohort are generally consistent with those reported in larger studies [3]. However, the prevalence of preoperative facial palsy (12.2%) highlights the potential for advanced diseases at presentation in some patients, underscoring the need for careful clinical and radiological assessment.

The accuracy of preoperative diagnostic procedures, particularly FNAC, remains a concern. In our study, FNAC correctly classified malignant parotid tumors in 51.6% of cases, with incorrect diagnoses in 38.7% and non-diagnostic results in 9.7%. This performance is consistent with the wide variability reported in larger series, where FNAC accuracy for parotid carcinoma has been estimated around 51–62% even in experienced centers. In a focused cohort of mucoepidermoid carcinomas, Vasudevan et al. reported correct FNAC diagnoses in only 64% of cases and described several characteristic pitfalls, underscoring the difficulty of reliably recognizing low-grade lesions. Conversely, in an international expert panel, Johnson et al. showed that cytomorphologic grading of nonbasaloid SGCs on FNA can reach an accuracy of approximately 90% for the dichotomous distinction between low-and high-grade tumors, while intermediate-grade lesions remained the most challenging and least reproducible category. Taken together, these data and our own results confirm that FNAC, although useful for triaging patients and broadly distinguishing low- from high-risk disease, has important limitations for precise preoperative histotype and grade assignment in routine practice. This distinction is nevertheless crucial for therapeutic decision making, since low-grade tumors are generally managed more conservatively, whereas high-grade neoplasms require more radical treatment. Consequently, even when FNAC suggests benign disease, surgical removal and definitive histopathological examination remain mandatory. Finally, emerging molecular profiling techniques, such as cDNA microarray and other high-throughput transcriptomic approaches, may in the future contribute to more accurate preoperative classification and risk stratification of salivary gland tumors, potentially improving the selection of patients for elective neck treatment; at present, however, these methods remain investigational and cannot be recommended as standard tools for routine preoperative decision making [14,15,16,17,18].

According to our institutional practice, when FNAC is non-diagnostic or not clearly informative, a second-level FNAB, preferably performed under ultrasound guidance, is generally considered. If the nature of the lesion remains uncertain despite cytologic or histologic sampling, the extent of surgery is planned on the basis of tumor location and the overall risk profile, taking into account clinical and radiological features—including facial nerve dysfunction, rapid tumor growth, skin fixation or ulceration, and other signs suggestive of biologically aggressive disease—rather than cytology alone. For lesions involving the superficial lobe, a superficial parotidectomy is usually performed, with careful identification and exposure of all facial nerve branches so that, if reoperation with total parotidectomy becomes necessary in the case of positive or close margins, the deep lobe can be safely addressed. For lesions arising from the deep lobe, total parotidectomy is typically performed upfront. In both scenarios, intraoperative frozen section may be used as a supportive tool to confirm malignancy and to refine the extent of parotid resection and neck management.

Our surgical management strategies, including partial, total, radical, and extended parotidectomy, reflect the diverse clinical scenarios encountered in parotid tumor surgery. The decision to perform neck dissection, either therapeutic or elective, was guided by clinical assessment and risk stratification. The relatively high rate of pN+ in the TND group (92.0%) confirms the appropriateness of this approach in patients with clinically evident nodal disease.

Our finding of a statistically significant correlation between tumor grade and nodal involvement (*p* < 0.05) aligns with the Westergaard-Nielsen et al. [19] study, which identified high-grade histology as a significant prognostic factor for reduced recurrence-free survival. While their study focused on overall recurrence, our data specifically links high-grade tumors to nodal metastasis, further emphasizing the importance of tumor grade in predicting disease behavior. However, it is important to note that grading is applicable only to certain histological types, as other subtypes exhibit an intrinsic biologic behavior, and attempted application of a universal grading scheme is merely a crude surrogate.

A key focus of our study was to investigate the role of elective neck dissection (END) in cN0 patients. While the overall rate of occult nodal metastasis in our cohort was 17.6%, this is similar to the 21% pooled proportion of occult metastases reported in the Westergaard-Nielsen et al. meta-analysis [20]. The rate varied significantly depending on histological subtype. Undifferentiated carcinoma and salivary duct carcinoma were associated with higher rates of nodal metastasis, while MEC was associated with a lower rate. Fisher’s exact test provides strong evidence of a statistically significant association between histological subtype and nodal metastasis in our data (*p* < 0.05).

Our analysis of disease-free survival in cN0 patients treated either with END or with observation does not reveal a statistically significant benefit associated with elective neck dissection (*p* > 0.05). (Figure 1). However, it is important to note that evaluating the clinical impact of this finding is challenging, as the patients were not selected randomly to undergo either neck dissection or observation, but on the contrary those who underwent END had already been selected based on various risk factors. Consequently, our findings are not consistent with recommendations for elective neck dissection in all cases, as suggested by Zbären et al. in their study [21]. In their study, where no strict criteria were used to select patients for different neck treatment modalities, it was demonstrated that END in cN0 patients reduces the risk of local recurrence compared to observation. In our study, we show that implementing preoperative stratification of patients to determine the necessity of neck dissection is effective, as it results in similar disease-free survival curves for both groups.

Interestingly, we found that all patients with intraparotid lymph node metastasis also had cervical nodal metastasis. Although our numbers are small, this consistent pattern suggests that intraparotid nodal involvement may act as a surrogate marker of advanced regional disease, rather than representing an isolated finding. From a clinical standpoint, this observation raises the hypothesis that intraoperative assessment of intraparotid nodes–for example through frozen section analysis–could be used to trigger an immediate selective neck dissection (END) in otherwise cN0 patients, thereby avoiding delayed or staged procedures. In practical terms, when intraparotid nodes are identified and appear suspicious during parotidectomy, their targeted excision and frozen section assessment might provide real-time information on occult cervical disease and support a more aggressive management of the neck in high-risk cases.

This novel finding warrants cautious interpretation and further investigation in larger, multicentre cohorts, but it is consistent with the growing recognition of the prognostic role of intraparotid lymph nodes. In particular, our results align with the study by Feng et al. [22], which highlighted the significance of intraglandular lymph node (IGLN) metastasis as an adverse prognostic factor in primary parotid cancer. Their series reported an IGLN metastasis rate of 32.9%, which is comparable to the 28.6% rate observed in our cohort. While Feng et al. did not specifically explore the correlation between intraparotid and cervical nodal metastasis, our observation that all patients with IGLN metastasis also harbored cervical nodal disease fits well with their broader conclusion that IGLN involvement is associated with poorer local control and worse prognosis. Taken together, these data support the concept that intraparotid metastasis should be regarded as a marker of biologically aggressive disease and may have important implications for tailoring neck management.

In our cohort, the analysis of disease-free survival according to nodal burden did not show a statistically significant difference between patients with 1–5 positive lymph nodes and those with more than 5 positive nodes (log-rank *p* > 0.05; Figure 3). However, this result must be interpreted with great caution, as only 14 patients had pathologically positive cervical nodes, leading to very small subgroups and limited statistical power. On the contrary, larger series (Aro et al.) [23] have consistently shown that increasing nodal burden is associated with worse outcomes in parotid carcinoma, and our Kaplan–Meier curves suggest a similar trend that fails to reach significance, most likely because of sample size constraints.

However, it is important to note that the Ketterer et al. study found that patients with any nodal metastasis (pN+) had significantly worse disease-free and overall survival compared to pN0 patients [24]. Our analysis of disease-free survival revealed a statistically significant difference between patients with pathologically negative lymph nodes (pN0) and those with pathologically positive lymph nodes (pN+), with a *p*-value < 0.05 (Figure 2). Nevertheless, in contrast to our study, the Ketterer et al. study did not find any occult metastases; every cN0 patient who underwent END was found to be pN0. This contrasts with our data and other data in the literature, and consequently, in that study, there was no difference in terms of DFS between cN0 patients undergoing END and those undergoing observation alone.

The Qian et al. study [25] on adenoid cystic carcinoma (ACC) found that the number of LNs removed was not associated with survival. Similar to the findings of Qian et al., our results showed no statistically significant evidence to suggest that the lymph node yield is associated with differences recurrences. However, we observed trends in odds ratios, such as higher relapse odds in Group A (0–17 LNY) vs. Group C (>40 LNY), which could warrant further investigation. This suggests that a larger dataset or the inclusion of additional variables might provide more insights into the potential impact of lymph node numbers on patient outcomes.

**Figure 4:**
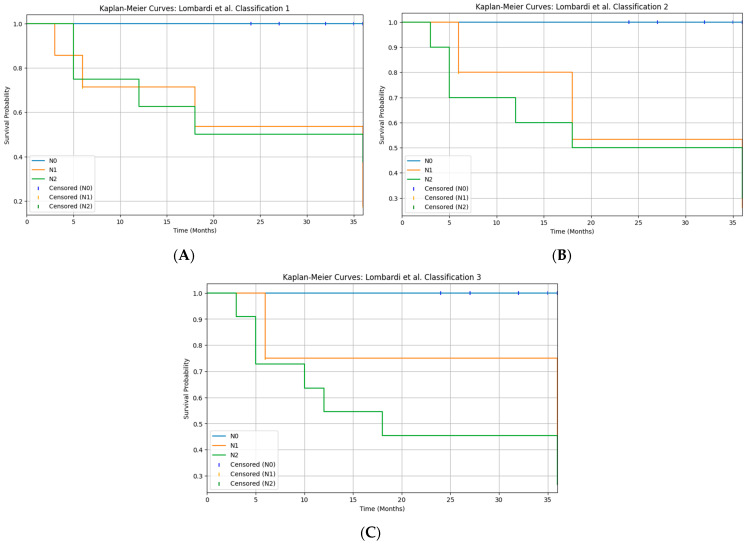
Kaplan–Meier curves for disease-free survival (DFS) according to the three Brescia N-classifications ((**A**): classification 1, number of metastatic lymph nodes; (**B**): classification 2, largest nodal metastasis; (**C**): classification 3, combined nodal burden). All three systems significantly stratify DFS (log-rank χ^2^ ≈ 16.9–18.7, *p* < 0.05).

The Lombardi et al. study [26,27] proposed three alternative N-classification systems for major salivary gland carcinoma, based on the overall number of metastatic nodes (classification 1), the largest diameter of metastatic nodes (classification 2), or a combination of both parameters (classification 3), showing that all three models provided better overall survival stratification than the current AJCC 8th N-categories in a large multicenter series. In the present study, we retrospectively re-staged our pN+ patients according to these three systems, including intraparotid nodes in the nodal count, and evaluated disease-free survival. All three N-classifications yielded a highly significant separation of DFS curves (log-rank χ^2^ 16.9–18.7; *p* < 0.001), and the combined burden model (classification 3), which defines “high nodal disease burden” as ≥4 metastatic nodes and/or at least one metastasis ≥20 mm, produced the most pronounced visual separation between risk groups (Figure 4). These findings, although based on a limited number of patients underwent neck dissection and therefore exploratory, support the concept that nodal disease burden—expressed as the number and/or size of metastatic nodes—has greater prognostic value than the current TNM N-categories, in agreement with both the original proposal and its external validation, where node-count-based staging consistently outperformed standard AJCC N-classification [28].

### Study Limitations

This study has several limitations. First, its retrospective, single-institution design without randomization may introduce selection bias and limits the generalizability of our findings. Second, the relatively small sample size (74 patients, 30 neck dissections, 14 pN+ cases) reduces the statistical power of subgroup analyses and increases the risk of type II error, particularly for outcomes related to nodal burden, lymph node yield and intraparotid metastasis. Third, the low number of patients with cervical nodal metastases did not allow a robust multivariable logistic regression for predictors of nodal involvement, and we therefore report only unadjusted associations. Fourth, although we fitted an exploratory multivariable Cox proportional hazards model for DFS including T-category, histologic grade and nodal status, the limited number of DFS events resulted in a suboptimal events-per-variable ratio; as a consequence, these adjusted hazard ratios should be interpreted with caution, and residual confounding by histologic subtype and other factors cannot be excluded. Taken together, these limitations indicate that our results should be regarded as hypothesis-generating and underscore the need for validation in larger, ideally multicenter, prospective cohorts.

## 5. Conclusions

In conclusion, despite the small sample size examined in our study, we believe that several important insights can be drawn regarding the management of malignant parotid tumors. First and foremost, nodal status (pN+ vs. pN0) is recognized as one of the most significant prognostic indicators in parotid carcinoma. Given the high likelihood of occult cervical metastases and regional recurrence [29], we advocate for elective neck dissection (END) to be performed in all cases of high-risk malignancies, particularly in locally advanced tumors (cT3-T4) exhibiting clinical facial nerve involvement, high-grade tumors, and certain histological types. Additionally, the implementation of frozen section analysis of intraparotid nodes during surgical intervention presents a promising approach to guide the decision-making process regarding the necessity for END [30]. Our findings also indicate that total parotidectomy should be strongly considered in cases where malignancy is detected preoperatively but grading remains uncertain, as this may reveal occult intraparotid metastases. These criteria are predicated on the presence of intraglandular lymph nodes that have a direct connection to the neck lymphatics in both the superficial and deep lobes, necessitating a more aggressive surgical approach to reduce the risk of relapse and loco-regional spread [31]. Furthermore, our data highlight a concerning lack of accuracy in fine-needle aspiration cytology (FNAC), reinforcing the notion that, even when FNAC results suggest benign lesions, surgical removal for comprehensive histopathological evaluation is essential. Ultimately, to personalize treatment strategies effectively, it is imperative to consider the entirety of the clinical and pathological characteristics inherent to each individual case, ensuring that management approaches are tailored to optimize patient outcomes.

To enhance the robustness and generalizability of our findings, future research should involve larger, multicenter cohorts with standardized data collection protocols. Such studies would allow for more precise stratification of patients based on tumor characteristics, treatment modalities, and outcomes, thereby refining the decision-making process for neck management in this complex patient population.

## Figures and Tables

**Table 1:** Preoperative diagnostic procedures and their performance in this cohort.

Modality	No. of Procedures, *n* (%) Among Patients with Preoperative Diagnosis *	Incorrect Diagnoses, *n* (%)	Nondiagnostic Results, *n* (%)
FNAC	31 (55.4%)	15 (48.4%)	3 (9.7%)
FNAB	5 (8.9%)	1 (20.0%)	2 (40.0%)
Open Biopsy	20 (35.7%)	-	-

* Preoperative histological or cytological assessment was performed in 56/74 patients (75.7%); percentages for each modality are calculated within this subgroup.

**Table 2:** This table summarizes the treatment modalities distribution.

Treatment Modality	Patients (*n*)	Patients (Rate)
Surgical treatment		
Partial parotidectomy	31	41.9%
Total parotidectomy	23	31.1%
Radical Parotidectomy	16	21.6%
Extended Parotidectomy	4	5.4%
Neck dissection		
Yes	30	40.5%
No	44	59.5%
Type of Neck dissection		
TND	13	17.6%
END	17	23.0%
Treatment modality		
Surgery alone	36	48.6%
Surgery and postoperative RT	15	20.3%
Surgery and postoperative RT-CHT	12	16.2%

CHT: Chemotherapy; END: Elective Neck Dissection; RT: Radiotherapy; TND: Therapeutic Neck Dissecti.

**Table 3:** This table summarizes patient distribution by histological types.

Histology	Absolute Number	Ratio
Acinic cell Carcinoma	6	8.1%
AdCC	7	9.5%
Adenocarcinoma	2	2.7%
Basal cell Adenocarcinoma	1	1.4%
Carcinoma ex pleomorphic	1	1.4%
Epithelial-Myoepithelial	4	5.4%
MEC	24	32.4%
Secretory Carcinoma	4	5.4%
Myoepithelial carcinoma	4	5.4%
Pleomorphic adenocarcinoma	0	0.0%
Salivary duct carcinoma	13	17.6%
SCC	6	8.1%
Undifferentiated Carcinoma	2	2.7%

**Table 4:** This table summarizes patient distribution by tumor grading.

Grading	Patients (*n*)	Patients (Rate)
High/intermediate	46	62.2%
Low	20	27.0%
Unknown/not specified	8	10.8%

**Table 5:** Patient breakdown based on pathological cancer features.

Pathological Features	Patients (*n*)	Patients (Rate)
T-classification		
T1/T2	48	64.9%
T3/T4	26	35.1%
N-classification		
N0	60	81.1%
N+	14	18.9%
Perineural invasion		
Yes	16	21.6%
No	58	78.4%
Lymphovascular invasion		
Yes	11	14.9%
No	63	85.1%

## Data Availability

The original contributions presented in this study are included in the article. Further inquiries can be directed to the corresponding author.

## Abbreviations

The following abbreviations are used in this manuscript:

ACC	Adenoid Cystic Carcinoma
AdCC	Adenoid Cystic Carcinoma
AJCC	American Joint Committee on Cancer
CHT	Chemotherapy
cN0	Clinically Node-Negative
CT	Computed Tomography
DFS	Disease-Free Survival
END	Elective Neck Dissection
FNAB	Fine-Needle Aspiration Biopsy
FNAC	Fine-Needle Aspiration Cytology
IGLN	Intraglandular Lymph Node
LN	Lymph Node
MEC	Mucoepidermoid Carcinoma
MRI	Magnetic Resonance Imaging
NCCN	National Comprehensive Cancer Network
OR	Odds Ratio
OS	Overall Survival
PET	Positron Emission Tomography
pN+	Pathologically Node-Positive
pN0	Pathologically Node-Negative
RT	Radiotherapy
RT-CHT	Radiotherapy plus Chemotherapy
SCC	Squamous Cell Carcinoma
SGC	Salivary Gland Cancer
SGT	Salivary Gland Tumor
TND	Therapeutic Neck Dissection
